# Polarization Beam Splitter Based on Si_3_N_4_/SiO_2_ Horizontal Slot Waveguides for On-Chip High-Power Applications

**DOI:** 10.3390/s20102862

**Published:** 2020-05-18

**Authors:** Yuxi Fang, Changjing Bao, Zhonghan Wang, Yange Liu, Lin Zhang, Hao Huang, Yongxiong Ren, Zhongqi Pan, Yang Yue

**Affiliations:** 1 Institute of Modern Optics, Nankai University, Tianjin 300350, China; 1120180104@mail.nankai.edu.cn (Y.F.); 1711143@mail.nankai.edu.cn (Z.W.); ygliu@nankai.edu.cn (Y.L.); 2 Department of Electrical Engineering, University of Southern California, Los Angeles, CA 90089, USA; changjib@usc.edu (C.B.); haoh@usc.edu (H.H.); yongxior@usc.edu (Y.R.); 3 School of Precision Instrument and Opto-Electronics Engineering, Tianjin University, Tianjin 300072, China; lin_zhang@tju.edu.cn; 4 Department of Electrical and Computer Engineering, University of Louisiana at Lafayette, Lafayette, LA 70504, USA; zpan@louisiana.edu

**Keywords:** optical waveguide, silicon photonics, silicon nitride, optical polarization

## Abstract

In this paper, we propose an Si_3_N_4_/SiO_2_ horizontal-slot-waveguide-based polarization beam splitter (PBS) with low nonlinearity for on-chip high-power systems. The coupling length ratio between the quasi-TE and quasi-TM modes (*L*_TE_/*L*_TM_) was optimized to 2 for an efficient polarization splitting. For the single-slot design, the coupling length of the PBS was 281.5 μm, while the extinction ratios (ER) of the quasi-TM and quasi-TE modes were 23.9 dB and 20.8 dB, respectively. Compared to PBS based on the Si_3_N_4_ strip waveguide, the coupling length became 22.6% shorter. The proposed PBSs also had a relatively good fabrication tolerance for an ER of >20 dB. For the multi-slot design, the coupling length of the PBS was 290.3 μm, while the corresponding ER of the two polarizations were 24.0 dB and 21.0 dB, respectively. Furthermore, we investigated the tradeoff between the ER and coupling length for the optimized PBSs with single slot or multiple slots.

## 1. Introduction

Photonic integrated circuit (PIC) has attracted much interest recently due to its fundamental advantages in terms of low power consumption, compact size, and low cost [1,2]. Specifically, silicon nitride (Si_3_N_4_) is considered to be a promising platform for PIC, due to its CMOS compatibility [3,4]. Compared to the silicon (Si) waveguide, Si_3_N_4_ has the advantages of a smaller roughness-induced scattering loss due to the lower refractive index, larger fabrication tolerance due to the weaker light confinement [5,6,7,8], and lower nonlinear loss induced by two-photon absorption (TPA) in the telecommunication band [9,10,11,12,13]. Thus, the Si_3_N_4_ waveguide has been widely considered for the high-power applications, such as nonlinear optics (optical frequency comb generation [14], chip optical parametric oscillators, supercontinuum generation [10]), material processing, laser medicine [15], etc.

Polarization beam splitter (PBS) is widely used to mitigate the polarization randomness problem in photonics systems by controlling the polarization state, thus, ensuring proper functioning [16,17,18,19,20]. PBS has been extensively used in sensor applications, such as polarization navigation sensor [21], photoacoustic remote sensing microscopy [22], distributed vibration sensor [23], magnetic field sensor [24], and fiber-optic gyroscope [25]. Furthermore, integrated PBS is an indispensable component of the transceiver in coherent optical communication system. The function of PBS is to separate the transverse electric (TE) and transverse magnetic (TM) polarization beams into different paths, due to their different propagation constants. Previously, various PBSs with different structures have been demonstrated, such as directional couplers, photonic crystals, multimode interferometers (MMIs), Mach-Zehnder interferometers (MZIs), and subwavelength gratings [16,26,27,28]. Recently, slot waveguide has been implemented into directional couplers for improved polarization-splitting performance [17,18,29,30,31,32,33,34,35]. In the slot waveguide, a low refractive index layer is sandwiched by two high refractive index layers. Due to the discontinuity of the electric field at the high-refractive-index-contrast interfaces, the mode with a polarization normal to the interfaces is significantly enhanced within the slot region. Either vertical-slot or horizontal-slot waveguides have been used for on-chip PBSs. The fabrication process of the vertical-slot-waveguide-based PBS usually introduces large roughness in the vertical interfaces [36]. A horizontal-slot-waveguide-based PBS has smoother interfaces, as it only needs to deposit layers with different materials and then etch the waveguide vertically.

In this study, we investigated the integrated PBS using Si_3_N_4_-based, coupled, horizontal slot waveguides that could be used for kW-level peak power on-chip systems. The designed Si_3_N_4_-based PBS could have negligible linear and nonlinear loss, and its Kerr nonlinear coefficient was approximately two orders of magnitude less than that of PBS using silicon waveguides. By manipulating the coupling length ratio of the quasi-TE and quasi-TM modes (*L*_TE_/*L*_TM_) to be around 2, efficient polarization splitting can be realized. First, we proposed and designed the Si_3_N_4_-based single-slot waveguide PBS, which had a 281.5-μm coupling length. The corresponding extinction ratios (ER) were 23.9 dB and 20.8 dB at 1550 nm for the quasi-TM and quasi-TE modes, respectively. Compared with the PBS based on Si_3_N_4_ strip waveguide, the coupling length becomes 22.6% shorter. Our simulation also showed good fabrication tolerance, the ER maintained >20 dB for the quasi-TM mode with a waveguide width variation of ±20 nm. Additionally, we designed a Si_3_N_4_-based multi-slot waveguide PBS with a 290.3-μm coupling length. Its ERs of the quasi-TM and quasi-TE modes were 24.0 dB and 21.0 dB at 1550 nm, respectively. Moreover, the tradeoff between the ER and coupling length for both the single-slot and multi-slot waveguides was also investigated at the end.

## 2. Concept

**Figure 1:**
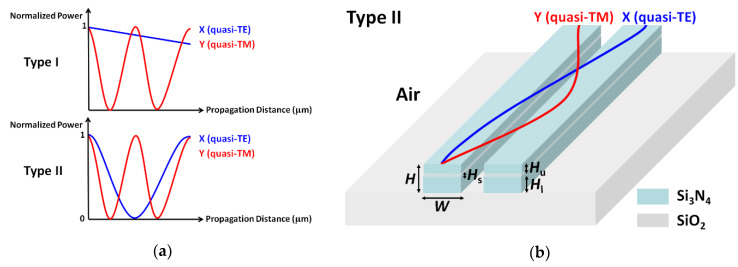
Schematic of two basic polarization splitting mechanisms (**a**) and the Si_3_N_4_ horizontal-single-slot-waveguide-based polarization beam splitter (PBS) (**b**).

Figure 1a shows two basic polarization splitting mechanisms. Type I illustrates that the coupling length of two orthogonal polarizations has a large difference, such as the coupling length ratio (*L*_TE_/*L*_TM_), which was up to 21 [18]. In this condition, most of the quasi-TE mode remained in the bar port, while a fraction of the quasi-TE mode was still coupled to the cross port. The quasi-TM mode was fully coupled to the cross port. The coupling length of the quasi-TE mode was designed to reach to the integer multiple of the one for the quasi-TM mode in Type II. In such a case, the quasi-TM mode was coupled out and then back to the bar port, while the quasi-TE mode was completely coupled to the cross port. As a result, an efficient polarization splitting was achieved. The schematic of the Si_3_N_4_ horizontal-single-slot-waveguide-based PBS is depicted in Figure 1b, which was based on Type II. The quasi-TE and quasi-TM modes had different coupling lengths when propagating along a waveguide. The blue and red lines represented the power transformation of the quasi-TE and quasi-TM mode, respectively.

The cross-section of the proposed Si_3_N_4_ horizontal-slot-waveguide-based PBS is also illustrated in Figure 1b. High index material Si_3_N_4_ was used for the top and bottom regions, while a low index silicon dioxide (SiO_2_) was chosen for the middle slot region. The material refractive indices of SiO_2_ and Si_3_N_4_ were obtained according to the Sellmeier equations in our model [37,38]. *H*_s_ is the slot thickness, *H*_u_ is the upper Si_3_N_4_ thickness, *H*_l_ is the lower Si_3_N_4_ thickness, and *d* is the spacing between the two slot waveguides. We keep the total height *H* and the width *W* of the slot waveguide to 800 nm and 1050 nm, respectively.

Based on the mode-coupling theory, the propagating behavior in the designed PBS could be viewed as the summation of the even and odd modes along the device. The coupling length of two orthogonal polarizations can be expressed as [18]:

*L*_TE_ = λ/[2*(*n*_e,TE_ − *n*_o,TE_)], (1)

**Figure 2:**
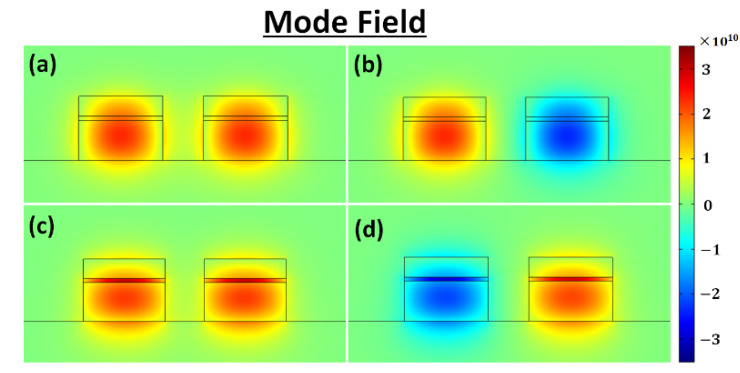
The electric field distributions of (**a**) the even quasi-transverse electric (TE) mode, (**b**) the odd quasi-TE mode, (**c**) the even quasi-TM mode, and (**d**) the odd quasi-TM mode.

*L*_TM_ = λ/[2*(*n*_e,TM_ − *n*_o,TM_)], (2)

where *L*_TE_ and *L*_TM_ are the coupling lengths of the quasi-TE and quasi-TM modes, *n*_e,TE_, *n*_o,TE_, *n*_e,TM_, and *n*_o,TM_ are the effective refractive indices of the even and odd supermodes of the *x* and *y* polarizations, respectively. We calculated the effective indices of the supermodes for the coupled-waveguide structure, using a finite-element mode solver. The electric field distributions of the quasi-TE (*n*_e,TE_ and *n*_o,TE_) and quasi-TM (*n*_e,TM_ and *n*_o,TM_) modes at the wavelength of 1550 nm are shown in Figure 2a–d.

## 3. Resultant Design of the Polarization Beam Splitter

### 3.1. Material Characteristics

We obtained the normalized power of the silicon slot waveguide [39] with different input peak power based on Equation (3)

**Figure 3:**
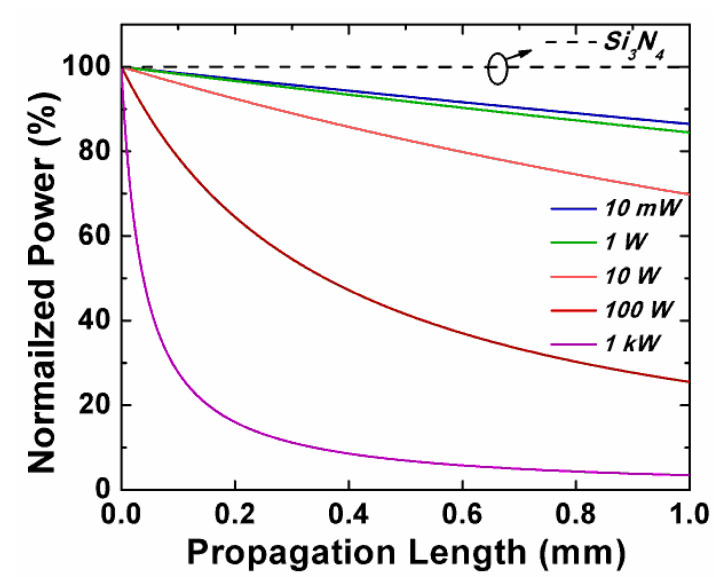
Normalized power of the inputs to the Si and Si_3_N_4_ horizontal slot waveguides.

*dI*/*dz* = −*αI* − *β_TPA_I*^2^, (3)

where *I* is the input peak power, *z* is the propagation distance, *α* is the linear loss, and *β*_TPA_ is the TPA coefficient. As per the literature results, 6.3 dB/cm [36] and 0.01 dB/cm [40,41] were chosen for the linear losses of the Si and Si_3_N_4_ slot waveguides, the TPA coefficient *β*_TPA_ of Si was set to 9.95 × 10^−12^ m/W [41]. In Figure 3, the normalized power of the silicon slot waveguide decreased rapidly as the increase of the input power to the waveguide due to nonlinear loss process. For high power on-chip applications, kW-level power was used in some experiments [42]. The normalized power only remained 27.8% after 0.1-mm propagation, when the input peak power was up to 1 kW. The dashed black line represented the power evolution in the Si_3_N_4_-based slot waveguide. In contrast, the normalized power after the 1-mm Si_3_N_4_-based slot waveguide still approached 99.98%. Consequently, compared to silicon horizontal slot waveguide splitter [18], it was more suitable for high power integrated systems.

**Figure 4:**
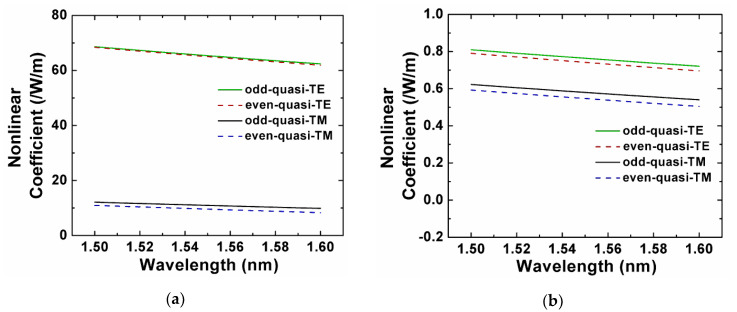
Nonlinear coefficient of the Si (**a**) and Si_3_N_4_ (**b**) horizontal slot waveguide PBS.

We used a full-vector model [43] to guarantee an accurate result, which considered the modal distribution of different materials to the overall nonlinear coefficient. Equation (4) was used to characterize the nonlinear coefficient:

(4) $$\gamma =2\pi {\overset{—}{n}}_{2}/\lambda A_{eff},$$

where *A_eff_* is the effective mode area, ${\overset{—}{n}}_{2}$ is the nonlinear refractive index averaged over an inhomogeneous cross-section weighted with respect to field distribution. At 1550 nm, the nonlinear refractive indices of Si, Si_3_N_4_, and SiO_2_ were 4.5 × 10^−18^ m^2^/W, 2.4 × 10^−19^ m^2^/W, and 2.6 × 10^−20^ m^2^/W, respectively [3,44,45]. The Kerr nonlinear coefficient *γ* of the silicon horizontal slot waveguide splitter [18] is shown in Figure 4a. The nonlinear coefficients of the quasi-TE modes were >68 /W/m, which were bigger than that of the quasi-TM modes. This was because the quasi-TE modes remained mainly in the silicon part, while a large portion of the optical power for the quasi-TM modes resided within the SiO_2_ slot part. Figure 4b shows the nonlinear coefficient of the modes in the proposed Si_3_N_4_ slot-waveguide-based PBS. The corresponding geometric parameters were *H* = 800 nm, *W* = 1050 nm, *H*_s_ = 50 nm, *H*_u_ = 250 nm, and *d* = 500 nm. We can see that the nonlinear coefficients of the quasi-TE modes were near 0.8 /W/m, the ones of the quasi-TM modes were near 0.6 /W/m. The Kerr nonlinear coefficients *γ* of the proposed PBS was approximately one percent of the one of the silicon horizontal slot waveguide splitter for the quasi-TE modes. For the quasi-TM modes, *γ* of the proposed PBS was near 5% of the one for the silicon horizontal slot waveguide splitter. Compared to the silicon horizontal slot waveguide splitter, the proposed Si_3_N_4_ slot-waveguide-based PBS had negligible nonlinearity under certain power level, and thus could efficiently separate the polarizations.

### 3.2. Single-Slot Waveguide Polarization Beam Splitter

**Figure 5:**
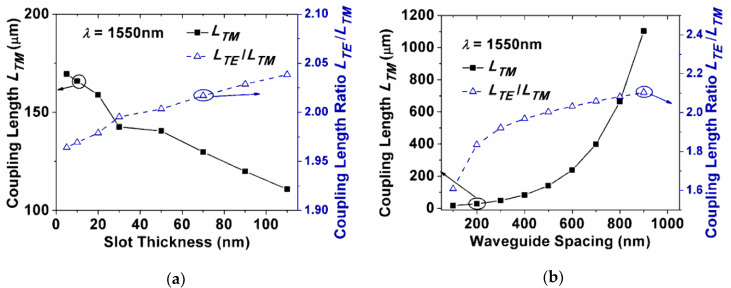
Coupling length of the quasi-TM mode and the coupling length ratio as a function of (**a**) the slot thickness *H*_s_ (*d* = 500 nm); and (**b**) the waveguide spacing *d* (*H*_s_ = 50 nm).

We first investigated the coupling length of the quasi-TM mode (*L*_TM_) and the coupling length ratio of quasi-TE and TM mode (*L*_TE_/*L*_TM_) by varying the slot thickness in the single-slot-waveguide-based PBS, as shown in Figure 5a. We found that the coupling length of the quasi-TM mode decreased with the increasing of slot thickness, and the coupling length ratio (*L*_TE_/*L*_TM_) increased. Here, the waveguide spacing *d* was 500 nm, and the slot thickness *H*_s_ was 50 nm. In this condition, the coupling length of the TM mode was 140.5 μm, and the coupling length ratio of the two polarizations approached to 2, thus, the two orthogonal polarizations could be effectively separated at 1550 nm. Figure 5b depicts the effects of varying the waveguide spacing *d*. For a PBS with 50-nm slot thickness, *L*_TM_ increases when *d* increases from 100 to 900 nm. The coupling becomes weaker for larger waveguide spacing, and thus the coupling length increases. The waveguide spacing *d* = 500 nm was chosen for the following study.

**Figure 6:**
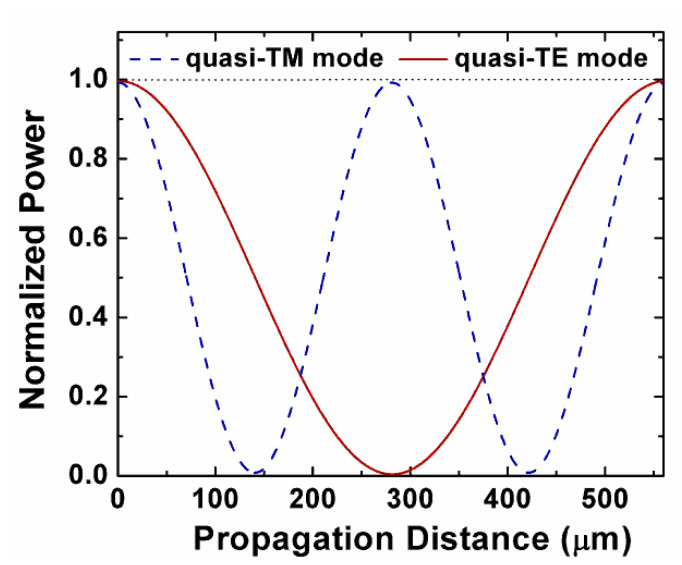
Normalized power exchange of the quasi-TM and quasi-TE modes at the bar port.

As shown in Figure 6, the normalized power of the quasi-TE and quasi-TM modes in the bar port was a function of propagation distance. When the propagation distance was 281.5 μm, the quasi-TE mode was almost completely coupled to the cross port, the residual output in the bar port was only ~0.4%. Furthermore, when the quasi-TM mode was coupled out and brought back to the bar port, the output remained at 99.22%. The device length *L* = *L*_TE_ = 2*L*_TM_ = 281.5 μm was chosen. Finally, an efficient PBS could be achieved by incorporating the coupled horizontal single-slot waveguides with the parameters of *H* = 800 nm, *W* = 1050 nm, *H*_s_ = 50 nm, *H*_u_ = 250 nm, *d* = 500 nm, and *L* = 281.5 μm.

Equations (5) and (6) are the general expression of the ERs:

*ER*_bar_ = 10*Log_10_[cos^2^(π*z*/2*L*_TE_)/cos^2^(π*z*/2*L*_TM_)], (5)

*ER*_cross_ = 10*Log_10_[sin^2^(π*z*/2*L*_TM_)/sin^2^(π*z*/2*L*_TE_)], (6)

where *ER*_bar_ and *ER*_cross_ are the extinction ratios of the bar port and the cross port of the PBS, respectively. These formulas are suitable for both the Type I and Type II polarization splitting mechanisms. Considering *L* = *L*_TE_ = 2*L*_TM_ in our Type II based design, the ERs in Equations (5) and (6) could be further expressed as:

*ER*_TM_ = 10*Log_10_[cos^2^(π*z*/*L*)/cos^2^(π*z*/2*L*)], (7)

**Figure 7:**
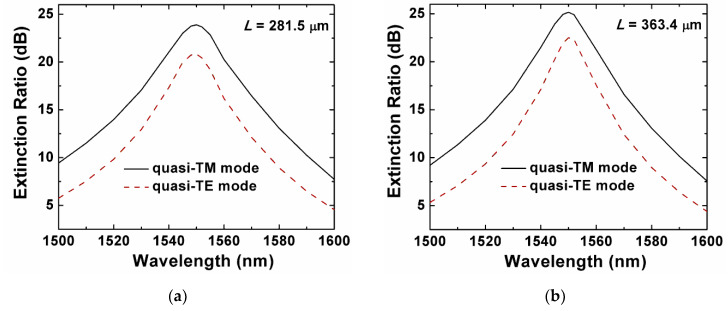
Extinction ratio of the quasi-TM mode at the bar port and the quasi-TE mode at the cross port of the proposed Si_3_N_4_ horizontal slot (**a**) and strip (**b**) waveguide PBS.

*ER*_TE_ = 10*Log_10_[sin^2^(π*z*/2*L*)/sin^2^(π*z*/*L*)], (8)

where *L* is the coupling length and *z* is the propagation distance. In the critical scenario, i.e., the beam propagation distance *z* = *L* = *L*_TE_ = 2*L*_TM_, the theoretical values of both *ER*_TM_ and *ER*_TE_ from the Equations (7) and (8) equaled to infinite. In practice, the fabrication imperfections from the design target, such as the length ratio of the two waveguides, the spacing between the two waveguides, and the width of the waveguides, resulted in incomplete power transfer and the degradation of the *ER*_TM_ and *ER*_TE_. We defined the bar port as the TM mode port, and the cross port as the quasi-TE mode port. Figure 7a shows the ERs of the proposed Si_3_N_4_ horizontal slot waveguide PBS for the quasi-TE and quasi-TM modes, which had a strong wavelength dependence. At the optimized wavelength of 1550 nm, the ERs of the quasi-TM and quasi-TE mode were 23.9 dB and 20.8 dB, respectively. The extinction ratio of the designed Si_3_N_4_/SiO_2_ horizontal-slot-waveguide-based PBS could almost satisfy the practical demand of optical communication systems within the total C-band [46]. The ER of the quasi-TM mode was larger than the one of the quasi-TE mode, as the electric field of the quasi-TE mode was discontinuous at vertical interfaces, the overlap between two waveguides was stronger, and the crosstalk was higher.

Furthermore, we investigated the corresponding Si_3_N_4_ strip-waveguide-based PBS and simulated its properties. Based on the same mechanism, efficient polarization splitting could be realized using the coupled Si_3_N_4_ strip-waveguide-based PBS. The Kerr nonlinear coefficients *γ* of the modes in the Si_3_N_4_/SiO_2_ slot waveguide was smaller than the ones in the Si_3_N_4_ strip waveguide for both polarizations. The optimized geometric parameters of the Si_3_N_4_ strip waveguide was *H* = 800 nm, *W* = 1070 nm, and the coupling length *L* = *L*_TE_ equaled 363.4 μm, when the corresponding ERs could achieve the values of the proposed PBS, i.e., 25.2 dB for the quasi-TM mode and 22.5 dB for the quasi-TE mode, respectively. Thus, the proposed Si_3_N_4_/SiO_2_ slot-waveguide-based PBS was improved on the properties for both coupling length and nonlinearity. As shown in Figure 7b, the ERs for the Si_3_N_4_-strip-waveguide-based PBS basically equaled to the ERs for the slot one in the wavelength range of 1500–1600 nm, which also had a relatively strong wavelength dependence. In comparison, the coupling length of the proposed Si_3_N_4_/SiO_2_ horizontal-slot-waveguide-based PBS was 22.6% shorter. Consequently, the Si_3_N_4_/SiO_2_ horizontal slot waveguide structure could effectively reduce the length of the elements.

**Figure 8:**
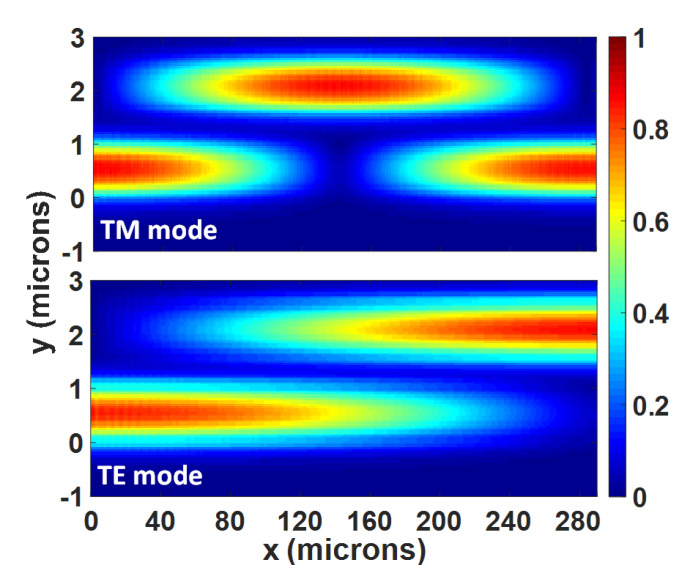
The power evolutions of the quasi-TM and quasi-TE modes in the designed PBS at 1550 nm.

We used the finite-difference time-domain method to simulate the modal evolution along the propagation of the proposed PBS. Figure 8 shows the power evolution along the propagation distance for the quasi-TE and quasi-TM modes at 1550 nm. We could observe that the optical power almost transferred from the bar port to the cross port at the coupling length (i.e., *L*_TE_) for the quasi-TE mode. Meanwhile, the quasi-TM mode was first transferred to the cross port when the propagation distance equaled to *L*_TM_ and then came back to the bar port. We could also observe that the coupling length of the quasi-TE polarization was twice as long as the one of the quasi-TM polarization.

**Figure 9:**
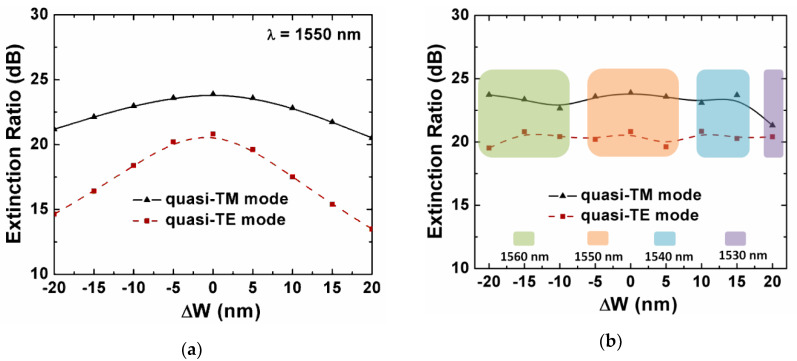
(**a**) Extinction ratio at 1550 nm of the quasi-TM and quasi-TE mode as a function of the waveguide width deviation Δ*w*; and (**b**) the maximum extinction ratio in the wavelength range of 1500–1600 nm of the quasi-TM and quasi-TE mode as a function of the waveguide width deviation Δ*w*.

We simulated the fabrication tolerance of the proposed Si_3_N_4_ horizontal slot-waveguide-based PBS. Taking into account the practical fabrication, the thickness of the slot and the strip thickness of the horizontal waveguide were defined by low-pressure chemical vapor deposition (LPCVD) or plasma-enhanced chemical vapor deposition (PECVD) [40]. These processes could be well controlled. The fabrication error was mainly from the etching process, i.e., the waveguide width was mainly the influence factor of the fabrication tolerance. Here, we defined the ∆*w* as the width deviation changing from −20 nm to +20 nm. When the waveguide width had an increase of ∆*w*, the waveguide spacing would decrease by a value of ∆*w*. As shown in Figure 9a, the ER maintained above 20 dB when ∆*w* varied from −20 to +20 nm for the quasi-TM mode at 1550 nm. For the quasi-TE mode, the fabrication tolerance was relatively low, and the ER dependence of the width deviation was more sensitive. The quasi-TE mode distribution was *x*-polarized, so the ER was more sensitive to the geometric parameters along the *x*-axis, such as the width of the waveguide *W*. In Figure 9b, we also simulated the maximum ER in the wavelength range of 1500–1600 nm, when the width deviation ∆*w* changed. The ER mainly fluctuated around the optimized value mentioned above, i.e., 23.9 dB for quasi-TM and 20.8 dB for quasi-TE. The center wavelength of the maximum ER drifted towards the shorter wavelength when the waveguide width increased by ∆*w*. Conversely, the center wavelength moved to a longer one as the waveguide width was smaller than the design target. Accordingly, we could adjust the waveguide width slightly to realize that the applications of the other central wavelengths in the C-band, without changing any other geometric parameter.

### 3.3. Multi-Slot Waveguide Polarization Beam Splitter

**Figure 10:**
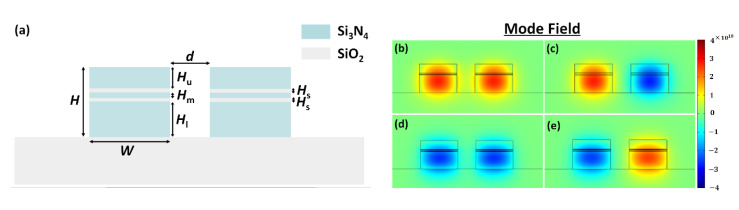
Schematic cross section (**a**) and the electric field distributions (**b**–**e**) of the multi-slot Si_3_N_4_-based waveguide PBS.

We also investigated coupled multiple-slotted waveguides for the polarization beam splitting. The schematic of the proposed multi-slot-waveguide-based PBS is shown in Figure 10a. Based on the optimized results of the single-slot waveguide, the multi-slot waveguide maintained the same parameters *H* = 800 nm, *W* = 1050 nm, *H*_u_ = 250 nm, and *d* = 500 nm. The slot structure was composed of two SiO_2_ layers with equal thicknesses, and the middle Si_3_N_4_ layer was embedded in between. Here, *H*_m_ was the thickness of the middle Si_3_N_4_ layer. The electric field distributions of the quasi-TE and quasi-TM modes of the multi-slot Si_3_N_4_-based waveguide PBS at the wavelength of 1550 nm are shown in Figure 10b–e.

**Figure 11:**
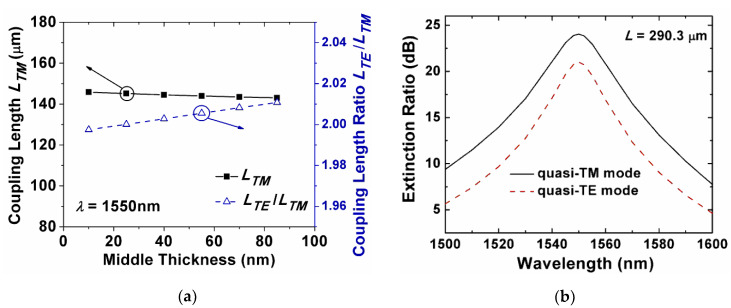
(**a**) Coupling length of the quasi-TM mode and the coupling length ratio as a function of the middle Si_3_N_4_ layer thickness *H*_m_ between the two SiO_2_ slots; and (**b**) the extinction ratio of the quasi-TM mode at the bar port and the quasi-TE mode at the cross port for the multi-slot-waveguide-based PBS.

In such a multi-slot waveguide, the thickness of the SiO_2_ and the inserted Si_3_N_4_ layers could determine the electromagnetic field distribution. The thicknesses of the two SiO_2_ slot layers were chosen as *H*_s_ = 20 nm, and the thickness of the middle Si_3_N_4_ layer was adjusted with a step of 15 nm. With the increase in the middle Si_3_N_4_ layer thickness, the power concentration was slightly decreased. The coupling between the two waveguides was, thus, increased. Consequently, the coupling length could be reduced as shown in Figure 11a. The middle Si_3_N_4_ layer thickness *H*_m_ was chosen to be 25 nm to keep the coupling length ratio of the quasi-TE and quasi-TM modes (*L*_TE_/*L*_TM_) close to 2. The corresponding coupling length *L* = *L*_TE_ equaled to 290.3 μm. Figure 11b shows the ERs of the quasi-TE and quasi-TM modes for the multi-slot-waveguide-based PBS from 1500 to 1600 nm. The ERs of the quasi-TM and quasi-TE modes were 24.0 dB and 21.0 dB at 1550 nm, respectively. We also investigated the fabrication tolerance of the multi-slot-waveguide-based PBS, the ERs were closed to the one based on the single-slot-waveguide for both modes at 1550 nm, as the width deviation ∆*w* changed from −20 nm to +20 nm. The wavelength dependence of the multi-slot PBS had a similar trend as the one of the single-slot PBS. From the previous research work, multiple slot waveguide could realize a higher confinement factor and power concentration [36,47], and further shorten the coupling length [48]. Compared with the single-slot-waveguide-based PBS, the properties of multi-slot-waveguide-based PBS did not show much improvement through our simulation, under the same overall size of the waveguides, materials, and simulation conditions, except for the number of slot layers.

### 3.4. Tradeoff between the ER and Coupling Length

**Figure 12:**
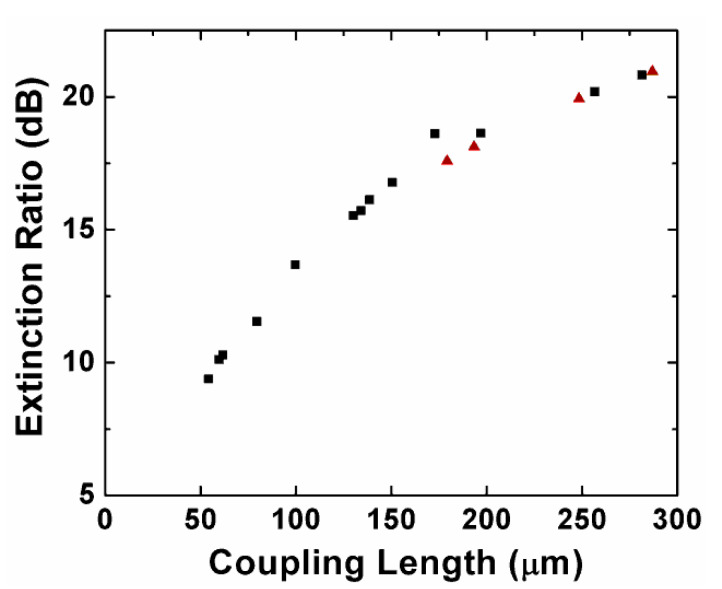
The tradeoff between the extinction ratios (ER) and coupling length of single-slot-waveguide-based PBS (square) and multi-slot-waveguide-based PBS (triangle).

Figure 12 shows the relationship between the ER and coupling length for the designed PBSs based on the Si_3_N_4_ single- and multi-slot waveguide. The square and triangle symbols represent single- and multi-slot-waveguide-based PBSs, respectively. In Table 1, single-slot-waveguide-based PBS with different parameters were used to investigate the polarization splitting effect. The table shows the relatively lower ER in the cross port (quasi-TE mode) at the wavelength of 1550 nm. The PBSs with the structure parameters in the table provide ~2 coupling length ratio (L_TE_/L_TM_), and the offset was within 0.5%. In Table 2, we list the different parameters of the multi-slot-waveguide-based PBS, based on similar conditions. One can see that both the single- and multiple-waveguide-based PBS showed a similar trend, such that although a shorter coupling length can be achieved by using smaller waveguide space or adjusting other parameters, the corresponding ER would be worse. In sum, we could obtain a shorter coupling length at the sacrifice of the ERs for both types of PBSs.

## 4. Conclusions

In summary, we presented Si_3_N_4_ single- and multi-slot-waveguide-based PBSs. Compared with Si PBS, Si_3_N_4_-based devices were more suitable for high-power integrated photonic applications due to its much lower Kerr nonlinearity and negligible nonlinear loss. Furthermore, we analyzed the effect of the geometric parameters and propagation length on the coupling length and ER. The coupling length of the proposed Si_3_N_4_/SiO_2_ horizontal-slot-waveguide-based PBS became 22.6% shorter than the one of coupled Si_3_N_4_ strip-waveguide-based PBS. The proposed structure could effectively reduce the length of the elements. The wavelength dependence of the quasi-TM and quasi-TE modes for these two designs were also investigated. The ERs for the quasi-TM and quasi-TE modes could both be >20 dB at 1550 nm for both the single-slot and multi-slot designs. The two designs showed good fabrication tolerance for up to ±20 nm width deviations. Finally, we studied the relationship between the ER and coupling length, and found that one needed to consider the tradeoff when optimizing the device performance or its size. To better serve the needs for different applications in on-chip high-power systems, one might introduce new design to further expand the operating bandwidth of the proposed PBS in the future.

## Figures and Tables

**Table 1:** Optimized Parameters of the Single-Slot Waveguide-Based PBS.

*H* (nm)	*W* (nm)	*H*_s_ (nm)	*H*_u_ (nm)	*d* (nm)	*L*_TE_/*L*_TM_	*L*_TE_ (μm)	*ER* (dB)
620	1200	100	260	200	2.0015	54.20	9.38
780	1150	100	410	200	2.0022	59.74	10.12
800	1150	90	410	200	1.9985	61.69	10.29
1200	1200	300	260	200	2.0040	79.48	11.54
840	1100	90	300	300	1.9995	99.75	13.68
950	1100	150	300	350	2.0001	130.14	15.53
1000	1100	150	340	350	1.9991	134.13	15.72
950	1050	150	410	400	1.9974	138.59	16.12
950	1050	120	440	400	1.9995	150.57	16.78
720	1000	90	250	500	1.9963	172.86	18.62
800	1000	90	390	500	2.0001	196.96	18.63
830	1050	90	250	500	2.0027	256.65	20.19
800	1050	50	250	500	2.0035	281.46	20.82

**Table 2:** Optimized Parameters of the Multi-Slot Waveguide-Based PBS.

*H* (nm)	*W* (nm)	*H*_s_ (nm)	*H*_u_ (nm)	*H*_m_ (nm)	*d* (nm)	*L*_TE_/*L*_TM_	*L*_TE_ (μm)	*ER* (dB)
840	1050	100	150	150	500	1.9990	179.29	17.58
840	1050	90	150	150	500	2.0041	193.41	18.12
800	1050	70	50	100	500	1.9966	248.44	19.93
830	1050	25	250	120	500	2.0017	286.89	20.95
